# Polymorphismsof the *CD24* Gene Are Associated with Risk of Multiple Sclerosis: A Meta-Analysis

**DOI:** 10.3390/ijms160612368

**Published:** 2015-06-01

**Authors:** Georgia G. Braliou, Katerina G. Pantavou, Panagiota I. Kontou, Pantelis G. Bagos

**Affiliations:** Department of Computer Science and Biomedical Informatics, University of Thessaly, Lamia 35100, Greece; E-Mails: gbraliou@dib.uth.gr (G.G.B.); kpantav@compgen.org (K.G.P.); pkontou@compgen.org (P.I.K.)

**Keywords:** *CD24*, meta-analysis, multiple sclerosis, predictive biomarkers, genetic association, genetic polymorphisms, genetic risk, protective variant

## Abstract

CD24 is a cell-surface protein mainly expressed in cells of the immune and central nervous system (CNS), cells that play a critical role in the development of multiple sclerosis (MS). In the current study, we investigated four polymorphisms of the *CD24* gene regarding their associations with MS. To this end, univariate and multivariate meta-analysis were applied along with modifications to include data from family-trios so as to increase the robustness of the meta-analysis. We found that the polymorphism 226 C>T (Ala57Val) of the *CD24* gene is associated with MS according to the recessive mode of inheritance (odds ratio = 1.75; 95% CI: 1.09, 2.81). Moreover, the 1527–1528 TG>del polymorphism is inversely associated with MS according to the dominant mode of inheritance (odds ratio = 0.57; 95% CI 0.39, 0.83). Conversely, the 1056 A>G and 1626 A>G polymorphisms were not found to be associated with MS. We conclude that the *CD24* 226 C>T polymorphism increases the risk of MS, while the 1527–1528 TG>del polymorphism seems to have a protective role against MS, suggesting that these two polymorphisms can be used as predictive biomarkers for MS development.

## 1. Introduction

Multiple sclerosis (MS) is a chronic and complex demyelinating disease of the central nervous system (CNS) with an occurrence of about 0.1% in Caucasian young adults [1]. The average age of onset of MS is between 20 and 40, and it is twice as common in women [2]. It is widely believed that the development of MS is attributed mainly to genetic susceptibility combined with environmental factors [3,4]. The most common genetic variants that are known to date belong to HLA class II, are located in chromosome 6p21 and are in linkage disequilibrium, while a variant of HLA-A (class I) is known to confer protection from MS [5]. According to a number of genome wide association studies (GWAS), at least 29 additional genomic regions are suggested to be associated with MS susceptibility [5] (and references therein).

Of particular note, *CD24*, although not detected in GWAS, is a gene that has been investigated thoroughly in regard to its association with autoimmune diseases, including MS, with promising results. The *CD24* gene is located in the chromosomal region 6q21. Interestingly, the particular region (6q) has previously been suggested to be in linkage with MS [6,7]. The CD24 protein is a GPI-anchored cell surface glycoprotein abundantly expressed in a variety of hematopoietic cells such as T and B cells, macrophages, neutrophiles, eosinophils and dendritic cells [8,9,10]. *CD24* is also expressed in cells of the CNS such as neural and gaglion cells, astrocytes and microglia [8,9,10,11], cells involved in the pathogenesis of MS. When expressed in T-cells of the nervous system, *CD24* expression is required for T-cell homeostatic proliferation [10]. It has also been shown that CD24 is responsible for the local expansion of T cells after migration to the CNS and the development of experimental autoimmune encephalomyelitis (EAE) in mouse models [12,13].

Four polymorphisms of the *CD24* gene have been found to be implicated in the etiology of MS and various degenerative diseases [10]; these polymorphisms are: (a) a C-to-T substitution at nucleotide 226 resulting in a Ala57Val substitution; (b) a TG dinucleotide deletion at positions 1527–1528; (c) an A-to-G substitution at nucleotide 1056; and (d) an A-to-G substitution at nucleotide 1626. The later three polymorphisms are located in exon 2 in the 3ʹ UTR, a region that confers mRNA stability [14,15]. rs numbers have been assigned to these polymorphisms (rs52812045, rs3838646, rs1058818 and rs1058881 respectively); however, all correspond to the intronless *CD24* pseudogene, which is located in the chromosomal region Yq11. A number of case-control studies have been performed to investigate the putative association of *CD24* gene polymorphisms with MS development and progression, although the results are controversial. In the present study, we performed a complete meta-analysis of the currently available bibliographic data in order to decipher these associations and add statistical support to them.

## 2. Results

### 2.1. Characteristics of Studies

A total of 19 articles were retrieved from our literature search from which seven articles with eight studies were found to fulfill the eligibility criteria for the meta-analysis of the *CD24* 226 C>T polymorphism comprising 2085 patients and 2295 healthy controls (Table 1). One study enrolled both case/control samples and family trios [11]. The remaining studies were population-based. Only two studies, out of a total of 19 retrieved articles, were found to include data pertinent to the meta-analysis of the polymorphisms 1527–1528 TG>del, 1056 A>G and 1626 A>G. The total numbers of patients and controls were 377 and 648, respectively. The characteristics of each study are summarized in Table 2A,B. The alleles and the genotypes of cases and controls were recorded and calculated separately for each study (Supplementary Tables S1–S4). In all studies, MS diagnosis was based on either McDonald [16] or Poser criteria [17].

**Table 1:** Polymorphisms of the *CD24* gene explored in the present study for their association with multiple sclerosis.

Polymorphisms	Number of Studies	Cases	Controls
226 C>T (Ala57Val)	8	2085	2295
1527–1528 TG>del	2	377	648
1056 A>G	2	377	648
1626 A>G	2	377	648

**Table 2:** (**A**) Characteristics of studies included in the meta-analysis for the association of the 226 C>T polymorphism of the *CD24* gene with MS; (**B**) Characteristics of studies included in the meta-analysis for the association of the 1527–1528 TG>del, 1056 A>G and 1626 A>G polymorphisms of the *CD24* gene with MS.

Study (First Author/Ref.)	Year	Country	Race	Cases	Diagnostic Criteria	Controls	Diagnostic Criteria
(**A**)							
Zhou Q. [11]	2003	USA	Caucasian	242	McDonald criteria	207	Blood samples from the American Red Cross (Columbus, OH)
Cui Y.Z. [18]	2006	China	Asian	110	Not reported	83	Not reported
Goris A. [19]	2006	Belgium	Caucasian	334	Poser criteria	322	Unrelated controls
Goris A. [19]	2006	UK	Caucasian	846	Poser criteria	846	Unrelated controls
Otaegui D. [20]	2006	Spain	Caucasian	141	McDonald criteria (mean age: 42.9 ± 12.7, 61% women)	285	Blood samples from anonymous healthy donors from the Gipuzkoa blood bank. (60.4% women, mean age 46.2 ± 11.6)
Ronaghi M. [21]	2009	Iran	Caucasian	217	McDonald criteria	200	Healthy individuals
Gonzalez S.J. [22]	2011	Argentina	Caucasian	102	Poser criteria	205	Age and gender-matched controls
Kollaee A. [23]	2011	Iran	Caucasian	120	McDonald criteria (mean age: 38.2 ± 8.5 years, range: 21–61)	120	Age, sex, and geographically matched healthy volunteers (mean age: 37.2 ± 7.8 years, range: 22–65) with no history of autoimmune or inflammatory disorders
(**B**)							
Wang L. [15]	2007	USA	Caucasian	275	McDonald criteria	443	Age and gender-matched controls
Gonzalez S.J. [22]	2011	Argentina	Caucasian	102	Poser criteria	205	Age and gender-matched controls

### 2.2. 226 C>T (Ala57Val) Polymorphism of the CD24 Gene

**Figure 1:**
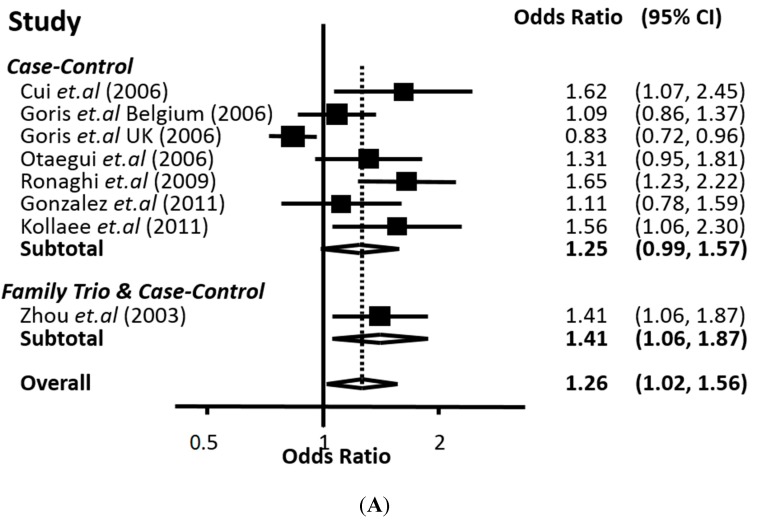

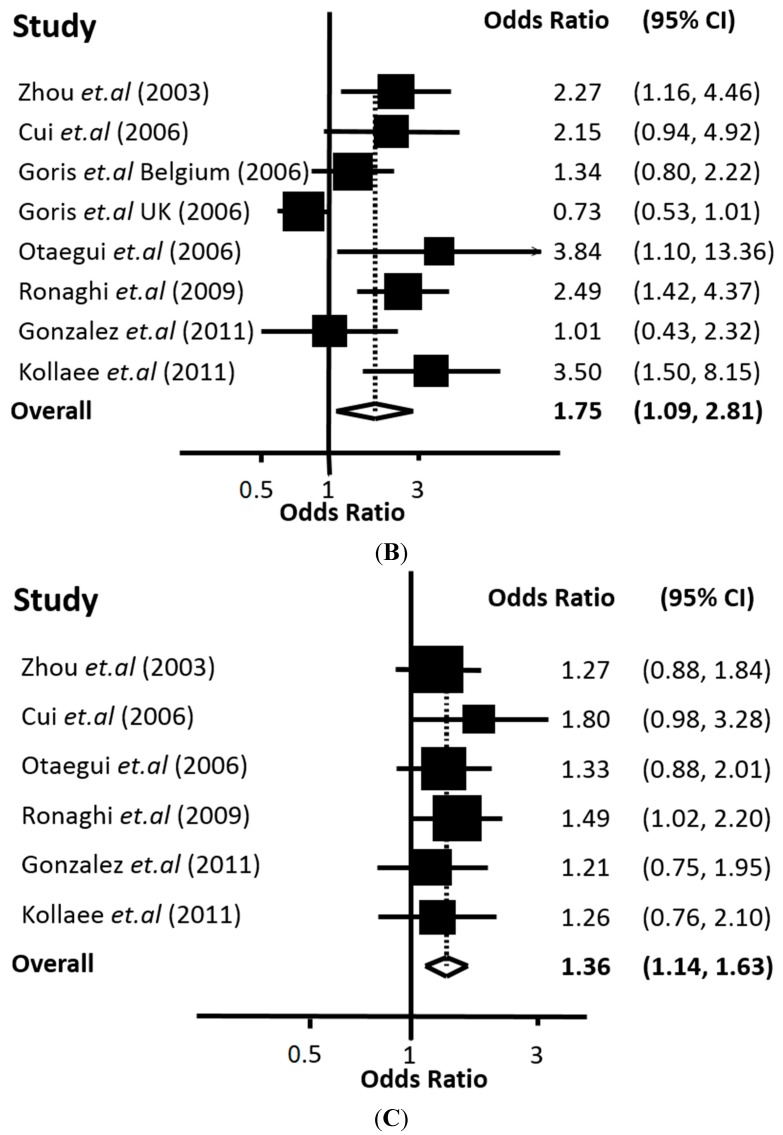
Forest plot of the meta-analysis for the association of the 226 C>T (Ala57Val) polymorphism of the *CD24* gene with multiple sclerosis. For each study the estimate of the variance, OR and its respective 95% Confidence Interval (CI) are plotted with a box and a horizontal line, respectively. The dashed vertical lines indicate the overall estimate, whereas the solid ones indicate the null effect (OR = 1). The ORs correspond to (**A**) the allele contrast T *vs.* C; (**B**) the TT *vs.* TC + CC contrast and (**C**) the TT + TC *vs.* CC genotype contrast.

The results of the eight populations analyzed in the present meta-analysis [11,18,19,20,21,22,23] suggested an association of the 226 C>T polymorphism with MS. The T *vs.* C contrast (incorporating data from family trios) produced a significant association with OR 1.26 and 95% CI: 1.02–1.56. Similarly, both recessive and dominant modes resulted in significant association (OR 1.75, 95% CI: 1.09–2.81 for TT *vs.* TC + CC and 1.36, 95% CI: 1.14–1.63 for TT + TC *vs.* CC) (Figure 1A–C). When the above contrasts were performed only with populations obeying Hardy Weinberg Equilibrium (HWE) the association remained significant (Table 3). Because two studies included data only for combined genotypes, only six studies were employed in the TT + TC *vs.* CC contrast. The allele and recessive contrasts presented higher heterogeneity compared to the dominant mode (Table 3). Time-trend and Proteus phenomenon were not detected in the cumulative meta-analysis for all contrasts. Sensitivity analysis (*i.e.*, removing a study and performing the meta-analysis again) showed that five out of the eight studies were necessary for achieving statistical significance, a finding that was expected considering the fact that the lower limit of the 95% confidence interval of the pooled estimate was close to unity. Nevertheless, the magnitude of the pooled OR did not change significantly in the sensitivity analysis (data not shown).

**Table 3:** Univariate meta-analysis for the 226 C>T (Ala57Val) polymorphism of the *CD24* gene with multiple sclerosis.

Contrast	Mode of Inheritance	Number of Studies	OR (Random Effects)	95% Confidence Interval (CI)	*I*^2^ (%)	Cochran’s *Q*	BSV ^a^	*Z*
T vs. C	Co-dominant	8	1.26	1.02–1.56	78.0	31.87	0.07	2.16
T vs. C in HWE	Co-dominant	4	1.39	1.17–1.66	0.0	2.33	0.00	3.70
TT vs. TC + CC	Recessive	8	1.75	1.09–2.81	76.9	30.25	0.33	2.34
TT vs. TC + CC in HWE	Recessive	4	2.05	1.27–3.31	32.1	4.42	0.08	2.93
TT + TC vs. CC	Dominant	6	1.36	1.14–1.63	0.0	1.50	0.00	3.34
TT + TC vs. CC in HWE	Dominant	4	1.32	1.05–1.67	0.0	1.21	0.00	2.33

^a^ BSV: Between Studies Variance.

To further elucidate the mode of inheritance, multivariate meta-analysis was performed on the combined data available from six studies. The contrast of risk homozygotes *vs.* wild-type allele homozygotes (TT *vs.* CC) yielded a robust and significant association with MS, having OR 2.44 and 95% CI: 1.77–3.38 (Table 4). Lambda (λ) was estimated to be equal to 0.18, indicating the recessive mode [24] as the prevalent mode of inheritance. The overall Wald test, employed to examine whether all ORs were equal to one (null hypothesis), suggested a definite association (*p*-value < 10^−4^).

**Table 4:** Multivariate meta-analysis for the 226 C>T (Ala57Val) polymorphism of the *CD24* gene with MS.

Contrast	Number of Studies	OR	95% CI
TC *vs.* CC	6	1.18	0.97–1.42
TT *vs.* CC	6	2.44	1.77–3.38

### 2.3. 1527–1528 TG>Del Polymorphism of CD24 

The potential association of the 1527–1528 TG>del polymorphism with MS was investigated using data from two independent studies [15,22]. A protective function of the del polymorphism (the TG dinucleotide deletion) against MS development was identified, since the allele contrast had OR smaller than unity and equal to 0.60 (95% CI: 0.41–0.85). The contrasts del/del *vs.* TG/TG + TG/del (recessive mode) yielded OR = 1.09 with 95% CI 0.06–18.64. Interestingly, the del/del + TG/del *vs.* TG/TG (dominant) gave a significant reverse association with OR = 0.57 with 95% CI 0.39–0.83 (Table 5).

Similarly, the TG/del *vs.* TG/TG contrast obtained under multivariate meta-analysis resulted in a strong association suggesting a protective effect of the polymorphism against MS (OR = 0.57 with 95% CI 0.38–0.87, Table 6). Dominance or even overdominance could be suggested since λ = 1.98 [24]. The overall Wald test further supported this association, given that the overall *p*-value was 0.038. Taken together, univariate and multivariate meta-analyses suggest that deletion of the TG at position 1527–1528 exerts a protective role against MS according to the dominant mode of inheritance.

**Table 5:** Univariate meta-analysis for the 1527–1528 TG>del polymorphism of the *CD24* gene with multiple sclerosis.

Contrast	Mode of Inheritance	Number of Studies	OR	95% CI	*I*^2^ (%)	Cochran’s *Q*	BSV ^a^	*Z*
del *vs*. TG	Co-dominant	2	0.60	0.41–0.80	0.0	0.55	0.00	2.8
del/del *vs*. TG/TG + TG/del	Recessive	2	1.09	0.06–18.64	55.2	2.23	0.00	0.06
del/del + TG/del *vs*. TG/TG	Dominant	2	0.57	0.39–0.83	0.0	0.2	0.00	2.89

^a^ BSV: Between Studies Variance.

**Table 6:** Multivariate meta-analysis for the 1527–1528 TG>del polymorphism of the *CD24* gene with MS.

Contrast	Number of Studies	OR	95% CI
TG/del *vs.* TG/TG	2	0.57	0.38–0.87
del/del *vs.* TG/TG	2	0.75	0.05–10.37

### 2.4. 1056 A>G and 1626 A>G Polymorphisms of CD24

Finally, meta-analyses were carried out for the 1056 A>G and 1626 A>G polymorphisms using data from two studies [15,22]. No significant association of the 1056 A>G polymorphism with MS was observed in all contrasts (allele and genotypes, Table 7). The heterogeneity was large (Table 7) while publication bias was also present (data not shown). The Barrowman *et al.* (2003) [25] method was applied to estimate the additional participants required for statistical significance. We found that 2059, 1901 and 2289 more participants would be required for the G *vs.* A, GG *vs.* GA + AA and GG + GA *vs.* AA contrasts, respectively, numbers that might not be very difficult to reach.

**Table 7:** Univariate meta-analysis for the 1056 A>G and 1626 A>G polymorphisms of the *CD24* gene with multiple sclerosis.

Polymorphism	Contrast	Number of Studies	OR	95% CI	*I*^2^ (%)	Cochran’s *Q*	BSV ^a^	*Z*
1056 A>G	G *vs.* A	2	1.70	0.68–4.23	96.0	24.96	0.42	1.13
	GG *vs.* GA + AA	2	2.23	0.58–8.61	95.5	22.26	0.91	1.16
	GG + GA *vs.* AA	2	1.59	0.69–3.62	86.3	7.32	0.31	1.09
1626 A>G	G *vs.* A	2	0.79	0.61–1.02	0.0	0.04	0.0	1.82
	GG *vs.* GA + AA	2	0.61	0.24 –1.58	0.0	0.12	0.0	1.02
	GG + GA *vs.* AA	2	0.78	0.58–1.04	0.0	0.08	0.0	1.71

^a^ BSV: Between Studies Variance.

Likewise, no statistically significant association was attained under any contrast for the 1626 A>G polymorphism with MS (Table 7). The heterogeneity was very low and publication bias was absent in all contrasts (data not shown). According to the Barrowman *et al.* (2003) method [25], statistically significant outcomes can be achieved with 164, 2760 and 322 more participants for the G *vs.* A, GG *vs.* GA + AA and GG + GA *vs.* AA contrasts, respectively, suggesting, as before, that statistical significance can be achieved easily.

## 3. Discussion

CD24 is a GPI-anchored cell membrane protein, with a molecular mass of 20 to 70 kD, and varying glycosylation. CD24 is a multifaceted protein implicated in inflammation, immunity—both adaptive and autoimmunity—and cancer [10]. Many experimental studies have focused on investigating the associations of *CD24* polymorphisms with autoimmune diseases such as MS, systemic lupus erythematosus (SLE), rheumatoid arthritis or giant cell arthritis [10] (and references therein). Although *CD24* was not detected in any GWASs, earlier classical linkage analyses [6] together with case control studies carried out in 2003 [11] suggested that *CD24* is a very promising biomarker for MS development. The fact that *CD24* was not identified in GWASs does not necessarily underestimate our study since GWASs often capture only a part of the phenotypic variance, due to intrinsic methodological limitations. The stringent criteria used for statistical significance in GWAS result, in many circumstances, in decreased power to identify a moderate effect. This happens because in the GWAS context, we usually work in an agnostic manner and the primary concern is to reduce the risk of spurious findings and false positive results arising from multiple testing. On the contrary, in the current context, we followed a hypothesis-driven approach. That is, the *CD24* gene had been chosen and studied in many independent studies due to some preliminary genetic evidence (*i.e.*, as mentioned, the linkage studies concerning the 6q region), as well as due to the presumed biological plausibility that might implicate CD24 antigen in the disease formation. Moreover, the present study includes populations from Iran and Argentina that were not included in previous GWASs. In support of the hypothesis that *CD24* plays a pivotal role in the development of autoimmune diseases, Li *et al.* (2006) [26] found that T cells undergo uncontrolled extensive proliferation in *CD24*-deficient mice. Thus, the homeostatic proliferation, which should be slow-paced, loses its self-limiting capacity, and this results in the rapid death of the recipient *CD24*^−^^/^^−^ mice.

The meta-analysis performed in this work, based on all the available data from the biomedical literature, clearly demonstrates a genetic association between the 226 C>T polymorphism of *CD24* with MS. Our conclusion is supported by two lines of evidence. First, with univariate meta-analysis, significant association was identified with all modes of inheritance (co-dominant, recessive and dominant). Second, from multivariate meta-analysis, which can indicate the mode of inheritance, the significant association was strengthened and the recessive mode appeared to be the most prominent. In any case, the association of the *CD24* 226 C>T polymorphism with MS proved to be quite robust according to the overall estimate of our analysis. The findings of our study are in accordance with previous case-control studies showing that the 226 C>T polymorphism of *CD24* is associated with another autoimmune disease, the SLE [27,28].

The idea that polymorphisms located in regulatory regions, can account for MS susceptibility has recently gained interest [29]. To this end, the 1527–1528 TG>del, 1056 A>G and 1626 A>G polymorphisms located in the 3′ UTR of the *CD24* gene were investigated. Our analysis showed that the 1527–1528 TG>del polymorphism has a protective role against the occurrence of MS. Significant associations were found in the allele and the del/del + TG/del *vs.* TG/TG genotype contrasts which both had zero heterogeneity. Multivariate meta-analysis further confirmed the above association and reinforced the dominant mode of inheritance. The same TG>del polymorphism has been shown in a case-control study to confer protection against SLE, as well [15]. Our findings are further corroborated by previous studies showing that this region [14] and especially the TG dinucleotide deletion confers instability to the *CD24* transcript [15], thus granting protection against MS and SLE onset and progression.

Conversely, based on meta-analyses we performed for the 1056 A>G and 1626 A>G *CD24* gene polymorphisms, no association was found under any contrast. Noticeably, all SNPs are in close chromosomal proximity to each other and one would expect Linkage Disequilibrium (LD) between them. The two associated polymorphisms with MS have a distance of about 1400 bp. Nevertheless, no definite LD has been observed between 226 C>T and 1527–1528 TG>del in the literature, neither for MS and SLE nor for other diseases such as Chron’s disease [15,30]. It is postulated that the *CD24* gene may have a recombination hotspot between these two polymorphisms [15]. Moreover, the fact that the other two SNPs, though located very close to the associated ones, are not associated with MS may suggest that the 226 C>T and the 1527–1528 TG>del polymorphisms play a causal role in MS, either promoting or preventive, respectively. A haplotype analysis, that would shed more light to this hypothesis, could not be performed in the present study due to lack of data in the included studies. Hence, it is clear that more studies (or collaborative meta-analyses of the current studies) investigating *CD24* haplotypes and their associations with MS are greatly needed to clarify the causative polymorphisms.

Despite the robustness of most of our results, the present study has some limitations. First, the number of studies included is relatively small, while the populations’ ethnic diversity is limited to Asians and Caucasians of European, USA, Argentinean and Iranian ancestry. Hence, additional studies with more participants from diverse ethnic backgrounds would be of great value, especially for the meta-analyses concerning the three polymorphisms located in the 3′ UTR. Second, significant heterogeneity was detected in three out of four analyzed polymorphisms. Regarding the 226 C>T polymorphism, heterogeneity was detected in the allele and the recessive mode contrasts. However, when the analyses were restricted to studies with populations in HWE, the heterogeneity was decreased. As far as the analysis of the 1527–1528 TG>del polymorphism is concerned, heterogeneity was present only in the recessive contrast, which yields non-significant results. Importantly, in the other contrasts, and particularly in the dominant mode contrast (the prevailing one) the heterogeneity was almost zero, reinforcing the robustness of the association. It is of particular interest that high heterogeneity appeared in all contrasts for the 1056 A>G polymorphism while zero heterogeneity was present in the contrasts of 1626 A>G polymorphism. Heterogeneity could be due mainly to small sample size, but also to the different criteria used to diagnose MS. Third, meta-analysis is subjected to methodological weaknesses of the original studies.

Overall, our study has several important advantages. First, a thorough and meticulous search was performed, including both a computer-aided and manual search, to identify all possible eligible studies included in published literature. Second, we did not impose any quality restriction and we did not exclude any study based on the design, the language or any other criteria, since most recent guidelines advocate against this. Third, we used modern statistical techniques in order to include all available studies (*i.e.*, family-based and population-based ones) and to detect the mode of inheritance. The multivariate method in particular is capable not only of identifying the mode of inheritance but also preserving the nominal type I error rate and protecting from multiple comparisons. Although the method identified the most plausible mode of inheritance, we chose to report all the available contrasts only for completeness. Finally, we took any available precaution in order to minimize different sources of bias, which is the main source of concern in this type of studies. Along these lines, we investigated the sources of heterogeneity, we searched for influential studies, and we performed all the available tests for detecting publication bias and time-trend bias.

In summary, we have conducted meta-analyses to investigate associations between *CD24* polymorphisms and MS using a method that combines data from case-control studies with family-based data [31]. We found that *CD24* 226 C>T polymorphism constitutes a risk of MS development while the dinucleotide TG deletion at 1527–1528 confers protection against the disease, and they can be used as prognostic biomarkers for MS onset. Any associations of 1056 A>G and 1626 A>G polymorphisms with MS could not be identified due to the small number of studies. We also estimated the number of the additional participants needed in order to reach significant results, using statistical methods. Given that these numbers can be attained relatively easily, biomedical researchers should be encouraged to investigate the association of the three 3′ UTR polymorphisms with MS and possibly with other autoimmune diseases. Furthermore, additional studies investigating linkage disequilibrium between these *CD24* polymorphisms, gene–gene interactions, or even gene-environment interactions would be helpful in better understanding the role of the *CD24* gene in MS onset and development.

## 4. Experimental Section

### 4.1. Literature Search

A comprehensive literature search was performed until September 2014 in PubMed (http://www.ncbi.nlm.nih.gov/pubmed) with the following keywords: “CD24 AND (gene OR variant OR polymorphism OR mutant OR mutation OR allele) AND (‘Multiple sclerosis’ OR MS OR ‘disseminated sclerosis’ OR ‘encephalomyelitis disseminate’)”. Retrieved abstracts were scrutinized and only the relevant ones were retained. The references from the retrieved articles were also investigated and the relevant ones were included in our study. We included all identified studies that provided data from which an estimate of the relative risk (the odds ratio) and its variance could be calculated. We did not impose any restrictions on the type of studies included with regard to the design, the language in which the study was written or any other quality measure.

### 4.2. Data Extraction

Data extraction from each manuscript was performed by two investigators (GGB, KGP) according to the eligibility criteria. In case problems of poor agreement occurred, they were resolved after discussion with the principle investigator (PGB) and the extracted data were recorded in a spreadsheet. From each article, the PubMed ID, first author’s name, year of publication, the total number of the subjects (cases/controls) as well as the population’s ethnicity and geographical location were recorded in the spreadsheet.

### 4.3. Statistical Analyses

Odds ratio (OR) was used to test the association between the mutant alleles and/or genotypes and MS, along with their 95% CIs (confidence intervals). In the case of zero cells, a continuity correction was applied by adding the value 0.5 to all cells of the contingency table. Data were analyzed using the random-effects method with inverse-variance weights [32]. The between-study heterogeneity was evaluated using the chi-square-based Cochran’s *Q* statistic and the consistency index (*I*^2^) [33]. The family-trio model was used when population studies included family trio data by applying the Transmission Disequilibrium Test and setting the affected offspring as “cases and non-affected parents as “controls” [31].

The multivariate random-effects method of meta-analysis, which can infer and quantify the genetic mode of inheritance directly, was also applied by estimating the ratio λ of the two log-odds ratios (of heterozygotes *vs.* homozygotes and of risk allele homozygotes *vs.* homozygotes for the wild type allele [24,34]).

To estimate possible publication biases, the rank correlation method of Begg and Mazumdar [35] was used. Additionally, the fixed effects regression method of Egger was applied [36]. Influential meta-analysis was further performed, by removing an individual study each time, and re-calculating the effects estimates (ORs) and heterogeneity. Cumulative meta-analysis was performed in order to estimate a possible time trend in the results over years, a bias called the “Proteus phenomenon” [37]. Two methods were used to detect the Proteus phenomenon: (a) the standard cumulative meta-analysis [38,39,40] approach; where we visually inspected the plot and (b) a more recently proposed regression-based method [37]. Finally, the Barrowman method [25] was used to estimate the additional participants required to achieve statistically significant results for the associations under study.

In all analyses, we used STATA 13 [41] and results with *p*-value <0.05 were considered statistically significant.

## Supplementary Materials

Supplementary materials can be found at http://www.mdpi.com/1422-0067/16/06/12368/s1.
