# Supplementary material for: Polymorphismsof the CD24 Gene Are Associated with Risk of Multiple Sclerosis: A Meta-Analysis
Source: Int J Mol Sci. 2015 Jun 1;16(6):12368–81. doi: 10.3390/ijms160612368 (PMC4490449; doi:10.3390/ijms160612368)
Supplement: Supplementary file 1 [file ijms-16-12368-s001.pdf]

Supplementary Information

Table S1. Studies reporting data on genotypes and alleles for the association of association of the 226C>T polymorphism of the *CD24* gene with MS.

Author	Year	Ethnicity	RACE	T [a]	NT [b]	Controls						Cases					
						TT	TC	CC	T	C	Total	TT	TC	CC	T	C	Total
Zhou Q.	2003	USA	Caucasian	13	2	13	85	109	111	303	207	32	97	113	161	323	242
Cui Y.Z.	2006	China	Asian			11	51	48	73	147	83	16	42	25	74	92	110
Goris A.Belgium	2006	Belgium	Caucasian			29			192	452	322	39			211	457	334
Goris A.UK	2006	UK	Caucasian			98			576	1116	846	74			509	1183	846
Otaegui D.	2006	Spain	Caucasian			4	136	145	144	426	285	7	69	59	83	187	141
Ronaghi M.	2009	Iran	Caucasian			20	66	114	106	294	200	47	68	102	162	272	217
Gonzalez S.J.	2011	Argentina	Caucasian			18	91	96	127	283	205	9	50	43	68	136	102
Kollaee A.	2011	Iran	Caucasian			8	49	63	65	175	120	24	40	56	88	152	120

[a] T: Transmitted; [b] NT: Non-transmitted.

Table S2. Studies reporting data on genotypes and alleles for the association of association of 1527–1528 TG>del polymorphism of CD24 gene with MS.

Author	Year	Ethnicity	RACE	Controls						Cases					
				TG/TG	TG/del	del/del	TG	del	Total	TG/TG	TG/del	del/del	TG	del	Total
Wang L	2007	USA	Caucasian	354	84	5	792	94	443	242	32	1	516	34	275
Gonzalez S.J.	2011	Argentina	Caucasian	182	23	0	387	24	205	94	7	1	195	9	102

Table S3. Studies reporting data on genotypes and alleles for the association of association of 1056 A>G polymorphism of CD24 gene with MS.

Author	Year	Ethnicity	RACE	Controls						Cases					
				A/A	A/G	G/G	A	G	Total	A/A	A/G	G/G	A	G	Total
Wang L	2007	USA	Caucasian	128	215	100	471	415	443	76	131	68	283	267	275
Gonzalez S.J.	2011	Argentina	Caucasian	61	103	41	225	185	205	26	58	94	110	246	102

Table S4. Studies reporting data on genotypes and alleles for the association of association of 1626 A>G polymorphism of CD24 gene with MS.

Author	Year	Ethnicity	RACE	Controls						Cases					
				A/A	A/G	G/G	A	G	Total	A/A	A/G	G/G	A	G	Total
Wang L	2007	USA	Caucasian	301	128	14	730	156	443	200	70	5	470	80	275
Gonzalez S.J.	2011	Argentina	Caucasian	160	43	2	363	47	205	94	18	1	206	20	102
